# Soluble amyloid precursor protein: a novel proliferation factor of adult progenitor cells of ectodermal and mesodermal origin

**DOI:** 10.1186/scrt77

**Published:** 2011-08-30

**Authors:** Michael P Demars, Amelia Bartholomew, Zuzana Strakova, Orly Lazarov

**Affiliations:** 1 Department of Anatomy and Cell Biology, The University of Illinois at Chicago, 808 S Wood St. Rm. 572 Chicago, IL 60612, USA; 2 Department of Surgery, The University of Illinois at Chicago, 840 S Wood St. Suite 402 Chicago, IL 60612, USA; 3 Department of Obstetrics and Gynecology College of Medicine, The University of Illinois at Chicago, 1801 W. Taylor Street, Chicago, IL 60612, USA

## Abstract

**Introduction:**

Soluble amyloid precursor protein α (sAPPα) is a proteolyte of APP cleavage by α-secretase. The significance of the cleavage and the physiological role of sAPPα are unknown. A crystal structure of a region of the amino terminal of sAPPα reveals a domain that is similar to cysteine-rich growth factors. While a previous study implicates sAPPα in the regulation of neural progenitor cell proliferation in the subventricular zone of adult mice, the ubiquitous expression of APP suggests that its role as a growth factor might be broader.

**Methods:**

sAPPα and α-secretase activities were determined in neural progenitor cells (NPCs), mesenchymal stem cells (MSC) and human decidua parietalis placenta stem cells (hdPSC). Inhibition of α-secretase was achieved by treatment with the matrixmetalloproteinase inhibitor GM6001, and proliferation was determined using clonogenic and immunocytochemical analysis of cell-lineage markers. Recovery of proliferation was achieved by supplementing GM6001-treated cells with recombinant soluble APPα. Expression of APP and its cellular localization in the subventricular zone was determined by Western blot and immunohistochemical analyses of APP wild type and knockout tissue. Alterations in pERK and pAKT expression as a function of soluble APPα production and activity in NPCs were determined by Western blot analysis.

**Results:**

Here we show that sAPPα is a proliferation factor of adult NPCs, MSCs and hdpPSC. Inhibition of α-secretase activity reduces proliferation of these stem cell populations in a dose-dependent manner. Stem cell proliferation can be recovered by the addition of sAPPα in a dose-dependent manner, but not of media depleted of sAPPα. Importantly, sAPPα operates independently of the prominent proliferation factors epidermal growth factor (EGF) and basic fibroblast growth factor (bFGF), but in association with ERK signaling and MAP-kinase signaling pathways. Levels of sAPPα and putative α-secretase, ADAM10, are particularly high in the subventricular zone of adult mice, suggesting a role for sAPPα in regulation of NPCs in this microenvironment.

**Conclusions:**

These results determine a physiological function for sAPPα and identify a new proliferation factor of progenitor cells of ectodermal and mesodermal origin. Further, our studies elucidate a potential pathway for sAPPα signaling through MAP kinase activation.

## Introduction

Amyloid precursor proteins (APPs) comprise a family of evolutionarily conserved single-pass type I transmembrane glycoproteins of an unknown physiological function. In mammals, that family includes APP and amyloid precursor-like protein 1 and 2 (APLP1 and APLP2) (reviewed in [1]). Mutations in *APP *cause familial Alzheimer's disease (reviewed in [1]). APP undergoes extensive enzymatic processing, producing both intracellular and extracellular metabolites (reviewed in [2]). In its non-amyloidogenic pathway, APP is cleaved mostly on the plasma membrane by an enzymatic activity termed the α-secretase [3,4]. α-Secretase cleaves APP between Lys16 and Leu17 of the Aβ region, resulting in the release of a soluble fragment (sAPPα) to the extracellular lumen and the retention of a membrane-tethered carboxyl-terminus fragment that undergoes further proteolysis (reviewed in [5]). The identity of α-secretase is not fully elucidated. Several enzymes-including members of the ADAM (a disintegrin and metalloproteinase) family ADAM10 [6], ADAM17 (TACE) [6-9], and ADAM9 as well as aspartyl protease beta-site APP-cleaving enzyme 2 (BACE2) [10]-are known to have α-secretase activity. Like APP, APLP2 is a substrate of ADAM10 and 17 [11]. Both ADAM10 and 17 are implicated in development-regulated notch signaling by ectodomain shedding of Notch ligands Delta and Jagged [12]. ADAM10 was recently suggested to be the main α-secretase in the brain [13].

sAPPα has been shown to exhibit neurotrophic and proliferative properties in fibroblasts [14], thyroid epithelial cells [15], and embryonic stem cells [16]. A crystal structure of a region of the amino terminal of sAPPα also reveals a domain that is similar to cysteine-rich growth factors, suggesting that sAPPα may act as a potential ligand for growth factor receptors [17]. Indeed, epidermal growth factor (EGF)-responsive neural progenitor cells (NPCs) in the subventricular zone (SVZ) have been shown to have binding sites for sAPP [18]. Deficiency of the sortilin-related receptor with type-A repeats (SORLA) results in enhancement of sAPP production, extracellular signal-regulated kinase (ERK) stimulation, and increased proliferation and survival of NPCs in both the SVZ and subgranular layer of the dentate gyrus (SGL) [19]. Taken together, these results suggest that APP may act as a growth factor in promoting cellular proliferation. However, there has been no follow-up to these studies. It is not clear what populations of NPCs sAPPα acts upon, whether it is a stand-alone factor or a co-factor, or whether it regulates non-neural adult stem cell populations.

Here, we show that the production of sAPPα by α-secretase processing of APP is an important event for the promotion of proliferation in a wide range of stem cell populations. We show, specifically, that sAPPα regulates the proliferation of NPCs, mesenchymal stem cells (MSCs), and Human decidua parietalis placenta stem cells (hdpPSCs). Importantly, sAPPα is a stand-alone proliferation factor, exerting its proliferative effect in an EGF- and basic fibroblast growth factor (bFGF)-independent manner. In the brain, levels of sAPPα are particularly high in the SVZ. In addition, we show that, in NPCs, sAPPα function is associated with the ERK signaling pathway. These results suggest that sAPPα is an essential proliferation factor for neural and non-neural adult stem cells. These observations further provide a functional significance for the abundance of APP.

## Materials and methods

### Primary neurosphere culture

Two-month-old C57/Bl6 wild-type mice were euthanized and their brains were removed and placed into sterile Dulbecco's modified Eagle's medium/F12 (DMEM/F12). A coronal slice (approximately 1 mm) was dissected starting 1 to 2 mm posterior to the olfactory bulb. The region occupying the lateral wall and anterior horn of the lateral ventricles was removed with the aid of a dissecting microscope and diced with a sterile scalpel. Neurosphere culture was prepared as previously described [20]. Briefly, tissue pieces were collected in a mixture of Papain and DNase in Earl's balanced salt solution and incubated at 37°C for 40 minutes. Then, tissue pieces were pelleted by centrifugation and dissociated to a single-cell suspension, and cells were plated in complete medium-water, DMEM/F12 (Gibco, now part of Invitrogen Corporation, Carlsbad, CA, USA), glucose (Sigma-Aldrich, St. Louis, MO, USA), NaHCO_3 _(Sigma-Aldrich), HEPES (Sigma-Aldrich), L-glutamine (Invitrogen Corporation), penicillin/streptomycin (Invitrogen Corporation), putrescine (9.6 μg/mL; Sigma-Aldrich), apotransferrin (0.1 mg/mL; Sigma-Aldrich), insulin (0.025 mg/mL; Roche, Indianapolis, IN, USA), selenium (5.2 ng/mL; Sigma-Aldrich), progesterone (6.3 ng/mL; Sigma-Aldrich), bovine serum albumin (BSA) (2 mg/mL; Sigma-Aldrich), heparin (4 μg/mL; Sigma-Aldrich), EGF (20 ng/mL; PeproTech, Rocky Hill, NJ, USA), and bFGF (10 ng/mL; PeproTech)-and passaged after 10 days.

### Isolation of mesenchymal stem cells

After euthanasia, the bone marrow contents of the femurs and tibia of donor Balb/C mice were flushed through a 40-μm filter (Becton, Dickinson and Company, Franklin Lakes, NJ, USA) into a 50-mL tube (Corning, Corning, NY, USA) containing MSC media: 40% alpha-modified Eagle's medium (Invitrogen Corporation, Rockville, MD, USA), 40% F-12 nutrient mixture (Invitrogen Corporation), 10% fetal bovine serum (Valley Biomedical, Winchester, VA, USA), and 1% antibiotic-antimycotic solution (Invitrogen Corporation). Bone marrow cells were plated at a density of 20 × 10^6 ^per 9.6 cm^2 ^in MSC media at 37°C in 5% CO_2 _as previously described [21]. The non-adherent population was removed after 72 hours, and the adherent cells were washed with fresh media and cultured for 7 days. The resulting adherent cells were harvested by incubating with 0.25% trypsin (Invitrogen Corporation) followed by gentle scraping. By means of negative selection via immunomagnetic column (Miltenyi Biotec, Auburn, CA, USA), cells negative for CD11b (eBioscience, San Diego, CA, USA) and CD45 (eBioscience) were placed back into culture. A homogenous cell phenotype was confirmed on the basis of the expression of CD29, CD44, and Sca1 and the absence of hematopoietic (CD45, CD14, and CD11b) markers. Prior to use, cells had been passaged from one to four times.

### Human decidua parietalis placenta stem cells

All studies were approved by the Institutional Review Board of the University of Illinois. hdpPSCs were isolated from the decidua parietalis dissected from placental membranes after normal vaginal delivery at term, as previously described in detail [22]. Human placenta tissue was obtained from the Human Female Reproductive Tissue bank in the Center for Women's Health and Reproduction at the University of Illinois at Chicago. Cells were cultured in RPMI-1640 medium supplemented with 10% heat-inactivated and charcoal-stripped fetal bovine serum, 0.1 mM sodium pyruvate, and 1% penicillin/streptomycin. At confluence, cells were trypsinized, propagated, and used for experiments in passage numbers three to five.

### Recombinant sAPP

sAPPα (Sigma-Aldrich) was used at 10 nM concentrations unless otherwise indicated (dissolved in phosphate-buffered saline, or PBS).

### Conditioned media

Neurosphere media was conditioned by plating 3 × 10^5 ^NPCs in each well of a 12-well plate in 500 μL of complete media. After 1 hour, media was removed and spun at 1,000*g *for 10 minutes to remove any cells or debris. For depletion of sAPP, conditioned media was precleared with protein A-agarose beads (Pierce, Rockford, IL, USA) and then incubated overnight at 4°C with 22C11 antibodies against the N-terminus of APP or IgG antibodies (Millipore Corporation, Billerica, MA, USA) as a control. Protein A-agarose beads were added for 30 minutes, the mixture was spun at 4,000 revolutions per minute (rpm) for 3 minutes, and the supernatant was used as depleted media. Regular conditioned media was subjected to the same process without antibody incubation as a control. All media was filtered through a 0.22-mm filter prior to addition.

### Detection of sAPP

For the detection of soluble APP from brain lysates, protein was extracted in immunoprecipitation buffer containing 150 mM NaCl, 50 mM Tris-Cl, 5 mM ethylenediaminetetraacetic acid (EDTA), 1% Triton-X 100, 0.5% sodium deoxycholate, protease inhibitor cocktail, and 250 μM phenylmethylsulfonyl fluoride (PMSF). To remove full-length APP, protein samples were immunodepleted by using 369 antibodies against the C-terminus of APP (a gift from Sangram S Sisodia, The University of Chicago). Briefly, samples were precleared with 50 μL of immobilized protein A-agarose beads (Pierce) at 4°C for 30 minutes. Samples were centrifuged at 4,000 rpm for 3 minutes, and the pellet was discarded. To the supernatant, 5 μL of 369 antibody was added and incubated overnight at 4°C. The next morning, 50 μL of immobilized protein A was again added for 30 minutes at 4°C and spun at 4,000 rpm for 3 minutes. The pellet contains the full-length APP-369 antibody complex, and the supernatant was probed for sAPP by using 22C11 antibodies raised against the N-terminus of APP (Millipore Corporation).

### Neurosphere formation (clonogenic) assay

Briefly, neurospheres were singly dissociated by mechanical dissociation and plated at 1,000 cells per well onto 96-well plates. For matrix-metalloproteinase (MMP) inhibitor experiments, cells were then treated with the indicated molar concentration of GM6001 or GM6001 negative control (Millipore Corporation) and the indicated molar concentrations of sAPP or conditioned media. If not otherwise indicated, 1 μM GM6001 and negative control inactive inhibitor (NC) were used. Cells were treated every 72 hours for 10 days. After 10 days in culture, neurospheres were counted under an inverted light microscope, and the average neurosphere diameter was calculated from 25 randomly assigned squares of the grid by using a Zeiss AX10 microscope (Carl Zeiss Ltd., Hertfordshire, UK) and StereoInvestigator software (MBF Bioscience, Williston, VT, USA). After sphere size determination, cells were singly dissociated with a p200 pipette and counted with a hemocytometer. The remaining cells were placed onto Matrigel-coated chamber slides for 30 minutes and then fixed in 4% paraformaldehyde for 30 minutes for immunocytochemistry. Briefly, cells were washed four times in Tris-buffered saline (TBS) and then placed into blocking solution (5% normal donkey serum, 0.25% Triton-X 100 in TBS) for 30 minutes at room temperature. Next, cells were incubated in primary antibodies-mouse anti-nestin (1:100; Millipore Corporation) and goat anti-Sox2 (1:200; Santa Cruz Biotechnology, Inc., Santa Cruz, CA, USA)-in TBS containing 0.25% Triton-X 100 for 1 hour at room temperature. After primary antibodies, cells were again incubated in blocking solution for 30 minutes at room temperature before secondary antibody incubation-anti-goat cy5 (1:250; Jackson ImmunoResearch Laboratories, Inc., West Grove, PA, USA) and anti-mouse cy3 (1:500; Jackson ImmunoResearch Laboratories, Inc.)-in TBS with 0.25% Triton-X 100 for 30 minutes at room temperature in the dark. Cells were then washed four times in TBS and incubated for 5 minutes with DAPI (4'-6-diamidino-2-phenylindole) (1:50,000; Invitrogen Corporation) at room temperature in the dark. Cells were then washed three times and mounted with polyvinyl alcohol-DABCO mounting solution. Cell counts were made by means of StereoInvestigator software version 8 (MBF Bioscience).

### Mesenchymal and placental cell proliferation experiments

MSC or hdPSC were trypsinized for 5 to 10 minutes with 0.05% trypsin, collected after trypsin inactivation, and spun at 500*g *for 5 minutes. Pellets were then dissociated and plated at 1,000 cells per well in 96-well plates. Cells were then treated with the indicated molar concentration of GM6001 or GM6001 negative control (Millipore Corporation) and the indicated molar concentrations of sAPP. After 3 days in culture, cells were trypsinized for 5 to 10 minutes, spun, and counted with a hemocytometer.

### Brain tissue for biochemistry and immunohistochemistry

Experiments using animals were performed according to guidelines of the National Institutes of Health and the University of Illinois at Chicago Institutional Animal Care and Use Committee. The *APP knockout *[*APP*(-/-)] model has been described previously [23,24]. Briefly, the authors generated the mice through homologous recombination in embryonic stem cells. Mice heterozygous for APP expression were cross-mated and *APP*(+/+) (*APP wild-type*), *APP*(+/-), and *APP*(-/-) resulted from this breeding. Our colony is maintained via group housing (fewer than five mice per cage) in a barrier facility under a 14:10 light/dark cycle with free access to food and water. Animal care and procedures were conducted according to the *National Institutes of Health Guide for the Care and Use of Laboratory Animals *[25].

### Brain tissue processing

For *in vivo *immunohistochemical staining, male *APP*(+/+) mice from 3 to 5 months old were used. All mice were anesthetized with a mixture of ketamine and xylazine and transcardially perfused with 100 mL of ice-cold PBS. The brains were then removed and halved in the sagittal plane. The left half was immediately placed into 4% paraformaldehyde on ice. From the right half of the brain, the following regions were dissected for biochemical analysis and immediately placed into Eppendorf tubes on dry ice: SVZ, hippocampus, olfactory bulb, frontal cortex, and cerebellum.

### Immunohistochemistry

Left hemibrains from PBS-perfused mice were post-fixed in 4% paraformaldehyde for 3 days and stored in 30% sucrose at 4°C. Hemibrains were sectioned sagittally at 50 μm by using a microtome and placed into cryopreservent (47.6% PBS, 28.57% ethylene glycol, and 25% glycerin vol/vol). Sections were blocked by using a solution containing 0.25% vol/vol Triton-X 100 (Sigma-Aldrich) and 5% vol/vol Normal Donkey Serum (Jackson ImmunoResearch Laboratories, Inc.) in TBS. The following antibodies were used: Dlx-2 (1:200; Millipore Corporation), nestin (1:100; Millipore Corporation), Sox2 (1:100; Santa Cruz Biotechnology, Inc.), ADAM10 (1:200; Millipore Corporation), and APP (22C11; Millipore Corporation and A8717; Sigma-Aldrich). Floating sections were incubated in primary antibodies for 72 hours at 4°C before continuing with blocking, biotin conjugation (Jackson ImmunoResearch Laboratories, Inc.), and secondary antibody incubation (cy2 Streptavidin, anti-mouse cy3, anti-goat cy5, and anti-rabbit cy5; Jackson ImmunoResearch Laboratories, Inc.).

### Western blotting

Protein extraction from brain tissue was performed in lysis buffer containing 1X TNE, 50 mM Tris, 150 mM NaCl, 5 mM EDTA, protease inhibitor cocktail (Sigma-Aldrich), and 100 mM PMSF. Quantification of protein was performed by using the bicinchoninic acid (BCA) method (Pierce), and equal amounts of protein were subjected to direct immunoblotting. For the extraction of protein from neurosphere, mesenchymal, and Human decidua parietalis placenta stem cells cultures, a lysis buffer containing 150 mM NaCl, 50 mM Tris-Cl, 5 mM EDTA, 1% Triton-X 100, 0.5% sodium deoxycholate, protease inhibitor cocktail, and 250 μM PMSF was used. For quantification, at least three cultures were used.

### Erk and Akt signaling

To assay phosphorylation of Erk and Akt, neurospheres were singly dissociated and plated at 5 × 10^5 ^cells per well in a six-well plate in Earle's balanced salt solution (Sigma-Aldrich) and treated immediately with 1 μM GM6001 or GM6001 negative control. After 1-hour incubation at 37°C, one of the GM6001-treated groups was subsequently treated with 10 nM recombinant sAPPα (Sigma-Aldrich) for 15 minutes before all groups were lysed in ROLB buffer: 10 mM HEPES, pH 7.4, 0.5% Triton X-100, 80 mM β-glycerophosphate, 50 mM sodium fluoride, 2 mM sodium orthovanadate, 100 nM staurosporine, 100 nM K252a, 50 nM okadaic acid, 50 nM microcystin, mammalian protease inhibitor cocktail (Sigma-Aldrich), and phosphatase inhibitor cocktail II (Calbiochem, now part of EMD Biosciences, Inc., San Diego, CA, USA) in water. After lysis, protein quantification was performed by using the BCA method (Pierce), and equal amounts of protein were run on Tris-glycine gels and transferred to nitrocellulose membranes. For blocking and antibodies, we employed a solution of 0.05% vol/vol Tween, 10% wt/vol milk, and 0.1% wt/vol BSA (Sigma-Aldrich) in TBS. The following antibodies were used: pErk (1:500; Santa Cruz Biotechnology, Inc.), Erk (1:1,000; Santa Cruz Biotechnology, Inc.), pAkt (1:1,000; Cell Signaling Technology, Inc., Danvers, MA, USA), and Akt (1:500; Cell Signaling Technology, Inc.). (N = 3 for Erk and Akt Western blot quantification.)

## Results

### sAPPα rescues proliferation of neural progenitor cells after treatment with a matrix-metalloproteinase and ADAM inhibitor

**Figure 1:**
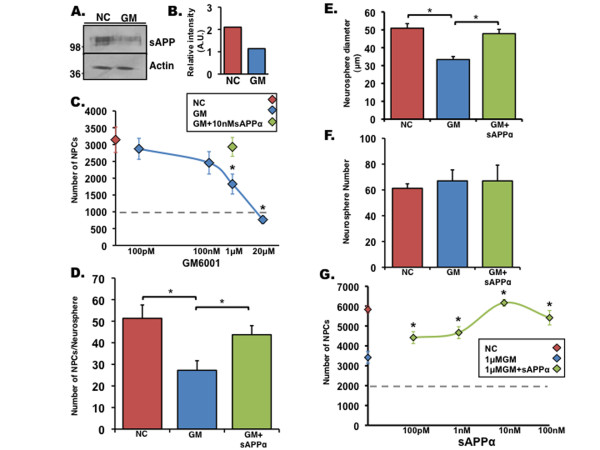
**Soluble amyloid precursor protein alpha (sAPPα) ameliorates matrix-metalloproteinase inhibitor-induced proliferation deficits in neural progenitor cells (NPCs)**. **(a) **Western blot analysis shows that levels of secreted sAPP are dramatically reduced in NPCs after treatment with matrix-metalloproteinase inhibitor GM6001 (GM) when compared with NPCs treated with an inactive inhibitor (NC). Actin from cell lysate was used as a control for protein load. **(b) **Optical density of expression intensity of sAPP normalized to actin. **(c) **NPC proliferation as assayed by a clonogenic assay. Total number of NPCs after 10 days in culture is reduced by GM6001 in a dose-dependent manner. Red diamond indicates 1 μM NC, blue diamonds indicate GM6001, green diamond indicates 1 μM GM6001 + 10 nM sAPPα, and dotted line indicates the number of cells originally plated. **(d) **Recombinant sAPPα (10 nM) can recover 1 μM GM6001-induced deficits in NPC proliferation as shown by a reduction in the number of NPCs counted per neurosphere observed in a clonogenic assay. **(e) **Measure of neurosphere diameter of neurospheres formed after a 10-day clonogenic assay in which NPCs were treated with 1 μM NC, 1 μM GM6001, or 1 μM GM6001 + 10 nM sAPPα. **(f) **The number of neurospheres formed after a 10-day clonogenic assay in which NPCs were treated with 1 μM NC, 1 μM GM6001, or 1 μM GM6001 + 10 nM sAPPα. **(g) **Dose response of sAPPα effect on NPC proliferation after inhibition with 1 μM GM6001 in a clonogenic assay. Red diamond indicates 1 μM NC, blue diamond indicates 1 μM GM6001, and green diamonds indicate recombinant sAPPα. Error bars represent standard error of the mean. *P < 0.05, analysis of variance with *post hoc *analysis. A.U., arbitrary units.

To examine the role of sAPPα in NPC proliferation and the significance of α-secretase cleavage of APP in this process, NPCs isolated from the SVZ of adult mice were treated with GM6001, a potent broad-spectrum hydroxamic acid-based inhibitor of MMPs and ADAMs which has been shown to decrease levels of sAPP and sAPLP2 [11]. First, using Western blot analysis, we confirmed that levels of sAPP are reduced in the conditioned media of NPCs treated with GM6001 (Figure 1a, b). Next, we singly dissociated NPCs and treated them with either inactive inhibitor (NC) or GM6001 or with GM6001 and sAPPα in a neurosphere formation assay. We observed that treatment of neurosphere culture with GM6001 significantly reduced proliferation of NPCs in a dose-dependent manner (Figure 1c). Inhibition of NPC proliferation could be rescued by the addition of recombinant sAPPα (Figure 1c), suggesting that sAPPα is sufficient for the induction of NPC proliferation. To further characterize the effect of sAPPα on NPC proliferation, we repeated the clonogenic assay, treated singly dissociated NPCs with NC or GM6001 or with GM6001 and sAPPα, and determined the number of neurospheres generated, their diameter, and the total number of NPCs (Figure 1d-f). We observed that, after treatment with GM6001, there were significant reductions in the diameter of neurospheres (Figure 1e) and the total number of NPCs observed in the cultures (Figure 1d) but not a significant reduction in the number of clones (Figure 1f), suggesting that treatment with GM6001 affects NPC proliferation without affecting clone formation capability. Likewise, treatment of neurosphere cultures with GM6001 and sAPPα significantly rescued impaired neurosphere diameter and total number of NPCs without affecting the number of neurospheres (Figure 1d-f). To determine whether recovery of NPC proliferation by sAPPα occurs in a dose-dependent manner, sAPPα was added to neurosphere culture at a concentration range of 100 pM to 100 nM. sAPPα recovered NPC proliferation after GM6001 treatment in a concentration as low as 100 pM. Rescue of NPC proliferation increased as a function of sAPPα dose and was maximal at 10 nM (Figure 1g). Taken together, these data suggest that α-secretase cleavage of APP is an important event in NPC cell cycle control and that sAPPα regulates NPC proliferation.

**Figure 2:**
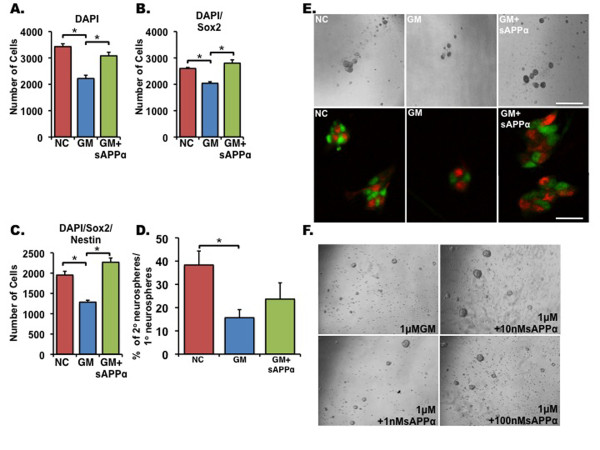
**Soluble amyloid precursor protein alpha (sAPPα) acts on both neural stem cells and neural progenitor cells to enhance proliferation**. Singly dissociated cells derived from the neurosphere formation assay described in Figure 1 were immunostained for DAPI **(a)**, Sox2 **(b)**, and Sox2/nestin **(c,e)**. GM6001 (GM) not only reduced total cell number but significantly reduced populations of Sox2 and Sox2/nestin-expressing cells. sAPPα rescued the total cell number by enhancing the proliferation of these populations (c). Representative images of Sox2/nestin double-labeling are shown (e). **(d) **The effect of GM6001 on neural stem cell self-renewal. Primary neurospheres were dissociated, and a single cell per well was cultured in a 96-well plate in the presence of NC, GM, or GM + sAPPα. The percentage of single cells that formed secondary neurospheres is shown. Scale bars = 85 μm (upper panel), 50 μm (lower panel). **(f) **Representative images of neurosphere formation assay showing the effect of varying concentrations of sAPPα on the size of GM6001-treated neurospheres. Error bars represent standard error of the mean. *P < 0.05, analysis of variance with *post hoc *analysis. DAPI, 4'-6-diamidino-2-phenylindole.

To determine the subpopulation of NPCs affected by GM6001 inhibition and recovered by sAPPα, singly dissociated NPCs treated with GM6001 or GM6001 + sAPPα were allowed to form neurospheres in a clonogenic assay as before and then immunolabeled with antibodies raised against nestin, an intermediate filament protein expressed in stem and progenitor cells [26], and Sox2, a transcription factor expressed in neural stem cells (NSCs) and NPCs of the adult brain [27]. We show that the number of nestin^+^Sox2^+ ^cells was significantly reduced after treatment with GM6001, and their number was fully recovered after addition of sAPPα (Figure 2a-c, 2e, f), suggesting that sAPPα affects the proliferation of both NSCs and NPCs. Thus, we proceeded to examine whether sAPPα affects self-renewal of NSCs (Figure 2d). For this purpose, secondary neurospheres were singly dissociated and cultured at one cell per well in 96-well plates, and the number of cells that generated tertiary neurospheres was quantified. We show that treatment of cells with GM6001 significantly reduced the number of generated neurospheres but that the addition of sAPPα significantly enhanced the number of neurspheres generated (Figure 2d), suggesting that sAPPα regulates both NSC self-renewal and NPC proliferation.

### sAPPα regulates neural progenitor cell proliferation in an EGF- and bFGF-independent manner

**Figure 3:**
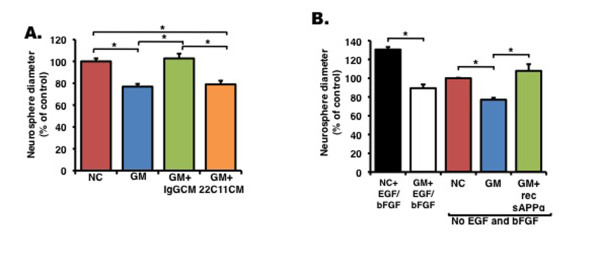
**Soluble amyloid precursor protein alpha (sAPPα) enhances proliferation in an EGF/bFGF-independent manner**. **(a) **Neural progenitor cell (NPC)-conditioned media that has been immunodepleted of sAPP with 22C11 antibodies fails to recover GM6001-induced proliferation deficits of NPCs, as assayed by neurosphere diameter in a neurosphere formation assay. In contrast, NPC-conditioned media immunodepleted with control IgG antibodies successfully recovers NPC proliferation. **(b) **A neurosphere formation assay was performed without growth factors (EGF and bFGF), and NPCs were treated with an inactive inhibitor (NC), GM6001 (GM), or GM6001 + recombinant sAPPα (GM+rec sAPPα). The proliferative effect of sAPPα on GM6001-treated neurosphere (GM) in medium devoid of EGF/bFGF and the proliferative effect of EGF/bFGF on GM6001-treated neurospheres are greater than the effect of EGF/bFGF on neurospheres treated with NC, suggesting a synergistic effect of sAPPα and EGF/bFGF. Error bars represent standard error of the mean. *P < 0.01, analysis of variance with *post hoc *analysis. bFGF, basic fibroblast growth factor; EGF, epidermal growth factor.

To address whether sAPPα is a stand-alone proliferation factor or its proliferative effect depends on other growth factors, we first compared the ability of NPC-conditioned media containing sAPPα to rescue NPC proliferation with that of conditioned media depleted of sAPPα. We observed that conditioned media containing sAPPα successfully rescued NPC proliferation but that conditioned media depleted of sAPPα failed to do so (Figure 3a). Next, we excluded proliferation factors (namely, EGF and bFGF) from the proliferation medium of NPCs and examined the rate of proliferation of cells by measuring clone formation and size (Figure 3b). As expected, neurosphere proliferation was reduced without EGF and bFGF. Interestingly, treatment of these neurospheres with GM6001 further reduced the extent of proliferation, suggesting that factors aside from EGF and bFGF regulate neurosphere proliferation (Figure 3b). Importantly, the addition of soluble APPα recovered GM6001-induced deficits in proliferation even without EGF and bFGF, suggesting that sAPPα operates independently of EGF and bFGF (Figure 3b). To determine whether sAPPα operates synergistically to these growth factors in regulation of neurosphere proliferation, we quantified the effect of sAPPα in medium devoid of EGF/bFGF and supplemented with GM6001 as well as the effect of EGF/bFGF in a medium supplemented with GM6001 and compared the effect on neurosphere diameter with the effect of EGF/bFGF on neurosphere diameter in medium supplemented with the negative control for GM6001. We observed that the additive effect of sAPPα in medium devoid of EGF/bFGF and supplemented with GM6001 and the effect of EGF/bFGF in a medium supplemented with GM is greater than the effect of EGF/bFGF in medium supplemented with the negative control for GM, suggesting a synergistic effect of sAPPα, EGF, and bFGF (Figure 3b).

### sAPPα regulates the proliferation of adult stem cells of non-neural origin

**Figure 4:**
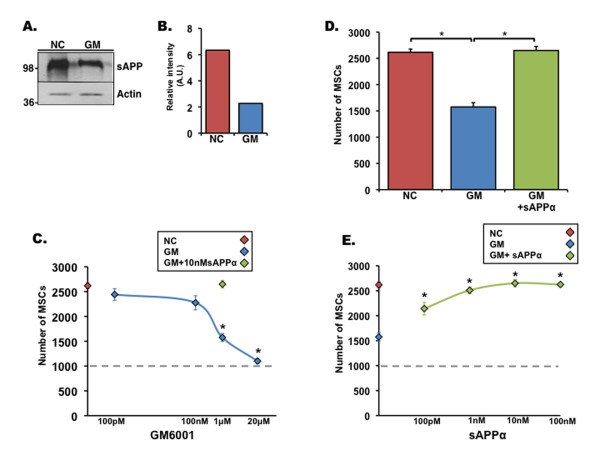
**Soluble amyloid precursor protein alpha (sAPPα) can recover matrix-metalloproteinase inhibitor-induced deficits in mesenchymal stem cell (MSC) proliferation**. **(a) **Western blot analysis shows that levels of secreted sAPP are dramatically reduced in MSCs after treatment with matrix-metalloproteinase inhibitor GM6001 (GM) when compared with MSCs treated with an inactive inhibitor (NC). Actin from cell lysates was used as a protein-loading control. **(b) **Optical density of expression intensity of sAPP (a) normalized to actin. **(c) **MSC proliferation as assayed by total cell number after 3 days in culture is significantly reduced by GM6001 in a dose-dependent manner. Red diamond indicates 1 μM NC, blue diamonds indicate GM6001, green diamond indicates 1 μM GM6001 + 10 nM sAPPα, and dotted line indicates the number of cells originally plated. **(d) **The number of MSCs counted after 3 days of 1 μM NC, 1 μM GM6001, or 1 μM GM6001 + 10 nM sAPPα treatment after original plating of 1,000 cells per well. **(e) **Dose response of sAPPα effect on proliferation of MSCs after 1 μM GM6001 addition. Red diamond indicates 1 μM NC, blue diamond indicates 1 μM GM6001, and green diamonds indicate recombinant sAPPα. Error bars represent standard error of the mean. *P < 0.01, analysis of variance with *post hoc *analysis. A.U., arbitrary units.

**Figure 5:**
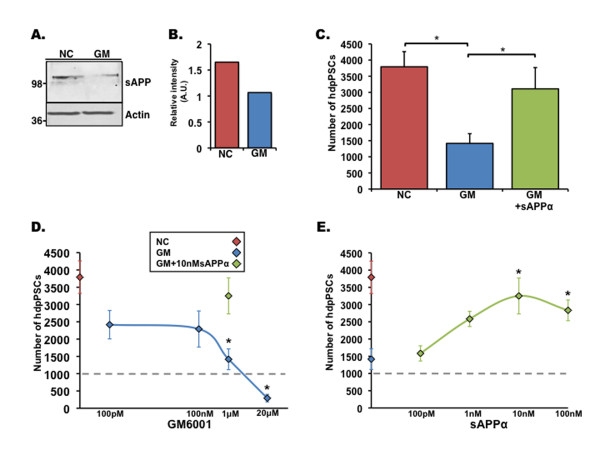
**Soluble amyloid precursor protein (sAPP) can ameliorate proliferation deficits in Human decidua parietalis placenta stem cells (hdpPSCs) induced by GM6001 (GM)**. **(a) **Western blot analysis shows that hdpPSCs treated with GM6001 secrete significantly less sAPP compared with hdpPSCs treated with an inactive control (NC). Actin serves as a protein-loading control. **(b) **Optical density representation of expression intensity of sAPP in Western blot shown in (a). **(c) **The number of hdpPSCs counted after 3 days of 1 μM NC, 1 μM GM6001, or 1 μM GM6001 + 10 nM sAPPα treatment after original plating of 1,000 cells per well. **(d) **hdpPSC proliferation as assayed by total cell number after 3 days in culture is reduced by GM6001 in a dose-dependent manner. Red diamond indicates 1 μM NC, blue diamonds indicate GM6001, green diamond indicates 1 μM GM6001 + 10 nM sAPPα, and dotted line indicates the number of cells originally plated. **(e) **Dose response of sAPPα effect on proliferation of hdpPSCs after 1 μM GM6001 addition. Red diamond indicates 1 μM NC, blue diamond indicates 1 μM GM6001, and green diamonds indicate recombinant sAPPα. Error bars represent standard error of the mean. *P < 0.05, analysis of variance with *post hoc *analysis. A.U., arbitrary units.

As APP is ubiquitously expressed and was shown to act upon fibroblasts and thyroid epithelial cells [14,15], we examined whether it regulates adult stem cells of non-neural origin. Thus, we first tested whether reduced proliferation after inhibition of MMPs and ADAMs was cell-autonomous to NPCs or applied to other stem cell types. For this purpose, we examined the extent of proliferation of MSCs after GM6001 treatment. As in NPCs, GM6001 treatment reduced secreted levels of sAPPα (Figure 4a, b) and significantly reduced proliferation of MSCs (Figure 4c) in a dose-dependent manner, suggesting that MMP family members may play an important role in the proliferation of MSCs. The addition of recombinant sAPPα significantly recovered MSC proliferation (Figure 4c, d). Like in the NPCs, 100 pM of sAPPα was sufficient to significantly rescue MSC proliferation and enhance their proliferation in a dose-dependent manner (Figure 4e). Taken together, these results suggest that APP is expressed in MSCs and processed by α-secretase activity to yield sAPPα that in turn regulates the proliferation of MSCs. Next, we investigated the effect of α-secretase inhibition and sAPPα on the proliferative activity of hdpPSCs. The effect of GM6001 on hdpPSCs was similar to the one observed with NPCs and MSCs (Figure 5). Treatment with GM6001 significantly reduced levels of secreted sAPPα concomitantly with hdpPSC proliferation (Figure 5a-c), and this deficit was ameliorated by the addition of recombinant sAPPα in a dose-dependent manner (Figure 5c-e). Taken together, these results suggest that sAPPα is an essential proliferation factor of a variety of adult stem cells of different origins and provides a functional significance for the abundance of APP.

### High levels of sAPP are expressed in the subventricular zone of adult mice

**Figure 6:**
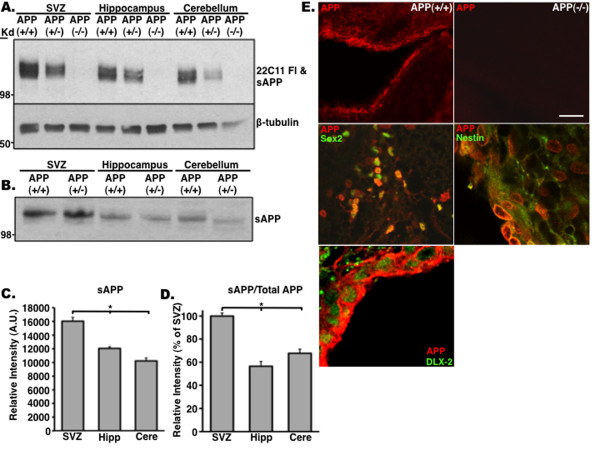
**Amyloid precursor protein (APP) is expressed in neural stem cells and neural progenitor cells of the adult subventricular zone (SVZ)**. **(a) **Western blot analysis shows higher expression levels of APP in the SVZ of *APP*(+/+) and *APP*(+/-) mice compared with the hippocampus and cerebellum. **(b) **Levels of sAPP are dramatically higher in the SVZ of *APP*(+/+) and *APP*(+/-) mice compared with the hippocampus and cerebellum. Optical density of expression level of APP **(c) **and sAPP **(d) **in Western blotting shown in (a). **(e) **Representative confocal images of APP staining in brain sections: Top panels: APP staining in the SVZ of *APP*(+/+) (left) and *APP*(-/-) (right) mice. Middle and bottom panels: High-power images of APP colocalization with Sox2 (middle left), nestin (middle right), and Dlx-2 (bottom) in the SVZ of *APP*(+/+) mice. Error bars represent standard error of the mean. *P < 0.009 (c), *P < 0.01 (d), analysis of variance with *post hoc *analysis. Scale bar = 250 μm. A.U., arbitrary units.

To establish that sAPPα plays a role in proliferation of NPCs *in vivo*, we examined expression levels of full-length APP and sAPPα in the SVZ, SGL, and a non-neurogenic region (cerebellum) of *APP*(-/-) and *APP*(+/+) mice. For this purpose, we used antibodies recognizing the N◯-terminus of APP (22C11), thus recognizing both full-length APP and sAPPα. We show that levels of 22C11 are comparable in the SGL and cerebellum with a trend of increased expression in the SVZ (Figure 6a). To examine whether this trend is a result of increased levels of sAPPα specifically, we immunodepleted full-length APP from the protein extract by using antibodies recognizing the carboxyl-terminus of APP, thus not capturing sAPPα. We observed that levels of sAPPα were particularly high in the SVZ of both *APP*(+/+) and *APP*(+/-) (Figure 6b-d). These results support our notion that sAPPα plays a major role in the SVZ.

To determine whether the NSC and NPC populations that express APP resemble those observed *in vitro *in neurosphere culture, we double-stained brain sections of adult mice with antibodies raised against APP, Sox2, nestin, and Dlx-2, the last of which is a homeobox protein expressed in transit-amplifying C cells of the adult SVZ [28]. APP expression colocalized with all three markers, suggesting that APP is expressed in NSCs and transit-amplifying NPCs in the adult SVZ (Figure 6e).

### ADAM10 is expressed in the subventricular zone and hippocampus of adult mice

**Figure 7:**
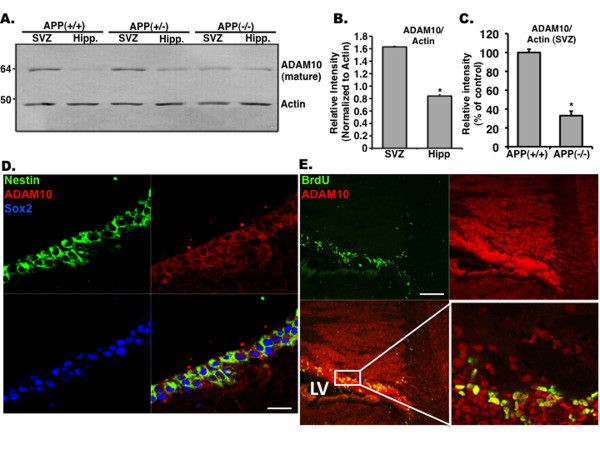
**Enhanced levels of ADAM10 in the subventricular zone (SVZ) of adult mice**. **(a) **Expression of the α-secretase ADAM10 is high in the SVZ compared with the hippocampus of *APP*(+/+) and *APP*(+/-) mice, suggesting a correlation with sAPPα levels. **(b) **Quantification of four Western blots showing higher ADAM10 levels in the SVZ compared with the hippocampus. **(c) **Quantification of four Western blots showing that ADAM10 expression is higher in APP(+/+) SVZ compared with that of the APP(-/-). **(d) **Representative images of ADAM10 colocalized with Sox2 and nestin in the adult SVZ. Scale bar = 125 μm. **(e) **Representative images of ADAM10 colocalized with BrdU after a single injection 24 hours prior to sacrifice. Error bars represent standard error of the mean. *P < 0.001 (b), *P < 0.01 (c), Student *t *test. Scale bar = 250 μm. ADAM, a disintegrin and metalloproteinase; APP, amyloid precursor protein; LV, lateral ventricle; sAPPα, soluble amyloid precursor protein alpha.

To examine whether high levels of sAPPα in the SVZ reflect levels of α-secretase in this region, we examined expression of ADAM10, recently shown to be a critical α-secretase during brain development [13]. We show that levels of ADAM10 are significantly higher in the SVZ compared with the SGL (Figure 7a, b). In support of a correlation between levels of sAPPα and ADAM10 is the observation that ADAM10 levels are lower in the SVZ of *APP*(-/-) compared with *APP*(+/+) (Figure 7a, c). To examine whether ADAM10 is expressed in the same neurogenic populations as APP, we immunostained brain sections with antibodies raised against ADAM10, nestin, Sox2, and BrdU 24 hours after a single dose. We observed that ADAM10 colocalizes with all three markers, suggesting that, like APP, ADAM10 is expressed in NSCs and transit-amplifying NPCs in the adult SVZ (Figure 7d, e).

### sAPP regulates extracellular signal-regulated kinase activity

**Figure 8:**
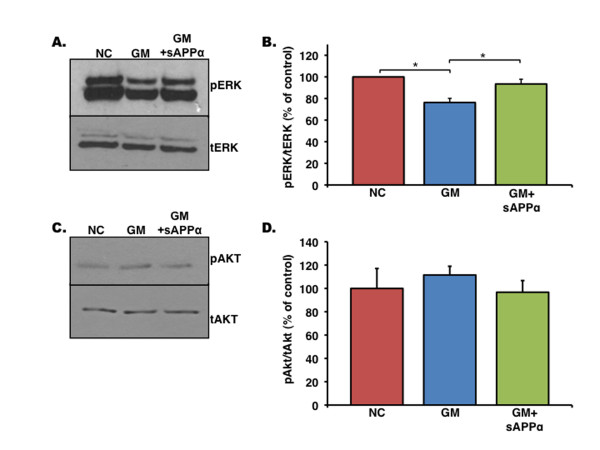
**Soluble amyloid precursor protein (sAPP) can recover GM6001 (GM)-induced deficits in Erk phosphorylation**. **(a) **Representative Western blot of neural progenitor cells (NPCs) treated with inactive inhibitor (NC), GM6001, or GM6001 + recombinant sAPPα and probed for pErk (top) and total Erk (bottom). **(b) **Quantification of pErk/total Erk Western blots. (*P < 0.05, n = 4, analysis of variance with *post hoc *analysis). **(c) **Representative Western blot of NPCs treated as described above and probed for pAkt (top) and total Akt (bottom). **(d) **Quantification of pAkt/total Akt Western blots (n = 4).

The mitogen-activated protein (MAP) kinase pathway plays an important role in the proliferation of NPCs derived from the adult SVZ [29]. Treatment with sAPP has been shown to stimulate ERK signaling in PC-12 cells [30], and SORLA-deficient mice showing enhanced APP cleavage to sAPP and Aβ display increased neurogenesis and ERK signaling in hippocampal neurons [19]. Therefore, we tested whether inhibition of MMP activity, decreasing sAPPα production, would result in deficient ERK signaling. For this purpose, NPCs were treated with GM6001 as before and placed into Hank's balanced salt solution for 1 hour to remove any presence of exogenous growth factor signaling. Western blot analysis reveals that pERK is reduced approximately 25% in NPCs treated with GM6001 (Figure 8a, b). To examine whether this reduction can be recovered by addition of sAPPα, we added recombinant sAPPα prior to lysing the cells. Recombinant sAPPα was able to reverse the deficits in ERK phosphorylation caused by GM6001 treatment (Figure 8a, b). To test whether this effect was specific to ERK, we examined another integral proliferation pathway known to respond to sAPPα [31], protein kinase B (Akt). Phosphorylation of Akt did not change significantly from negative control in any of the groups tested (Figure 8c, d), suggesting that sAPPα is associated specifically with ERK signaling.

## Discussion

During the last two decades, there has been much interest in unraveling the physiological role of APP, its metabolites, and the physiological significance of its multiple metabolic pathways. Here, we show that sAPPα, a product of APP cleavage by α-secretase, is a proliferation factor of NPCs in the adult brain, MSCs, and hdpPSCs. This suggests an important role for sAPPα as a proliferation factor of different stem cell populations in the adult. The diversity of stem cells that respond to sAPPα as a proliferation factor leads us to suggest that sAPPα exerts its effect on a wide range of stem cell populations. NPCs used in this study were derived from the adult mouse brain. MSCs were isolated from the bone marrow of adult mice, and hdpPSCs were isolated from the decidua parietalis dissected from placental membranes. All stem cell populations have self-renewal capability. Whereas NPCs are capable of differentiating into neurons or glia only, MSCs exhibit multilineage potential into many mesodermal cell types and possibly into endodermal and ectodermal cells as well (reviewed in [32]). hdpPSCs are of human fetal origin and are thought to exhibit multilineage potential [33].

These results provide functional significance for the fact that APP is ubiquitously expressed and is highly conserved in evolution (reviewed in [34]). We show that sAPPα is a potent factor that can recover proliferation of these stem cell populations after treatment with an MMP inhibitor. The importance of our observations is supported by studies reporting that the expression of sAPPα can rescue behavioral and physiological deficits of *APP *knockout mice, such as growth and brain weight deficits, agenesis of the corpus callosum, and impaired spatial learning associated with impaired long-term potentiation, suggesting that sAPPα is sufficient for the execution of APP function compromised in its absence [35,36].

APLP1 and APLP2 have substantial homology to APP, both within the ectodomain and particularly within the cytoplasmic tail [37]. Support for functional redundancy between APLP2 and APP/APLP1 comes from studies of combined knockouts [38]. Cleavage of APLP1 and APLP2 by α-secretase yields sAPLPα of unknown function, which may play a role similar to that of sAPPα in NPC and other stem cell populations. As we cannot exclude a potential functional redundancy between sAPPα and sAPLP2α, we avoid a potential compensatory effect of sAPLPα in the present study by using the GM6001 MMP inhibitor that inhibits APLP1,2 cleavage by α-secretase as well. Expression levels of sAPPα were particularly high in the SVZ. That may suggest that NPC proliferation is differentially regulated in the two neurogenic microenvironments in the adult brain [39] (and that sAPPα plays an important role in the SVZ specifically) or simply reflect the fact that the neurogenic population is larger in the SVZ. Expression levels of the α-secretase, ADAM10, are significantly higher in the SVZ compared with the dentate gyrus or to the SVZ of APP(-/-), suggesting a correlation between α-secretase and sAPPα in the neurogenic regions. Like in the case of sAPPα, the higher level of ADAM10 expression in the SVZ may be a reflection of the greater population of NSCs and NPCs residing in the SVZ. While GM6001 inhibited α-secretase activity in NPCs, MSCs, and hdpPSCs, we cannot exclude the possibility that the identity of the dominant α-secretase in each one of these stem cell populations is different, representing a different member of the ADAM family, each of which is differentially expressed in a tissue- or niche-specific manner.

Finally, we provide an insight into the mechanism by which sAPPα exerts its proliferative effect in NPCs. We show that it acts in an EGF- and bFGF-independent manner. It has been suggested that, because of the presence of a heparin-binding domain adjacent to the putative growth factor domain in APP [40] and the presence of sAPP-binding sites on EGF-responsive NPCs [18], signaling of sAPP may involve other factors (such as EGF) which also can interact with heparin. Our results suggest that any interaction of sAPPα and EGF or bFGF is not necessary for the proliferation-related functions of the molecule. Furthermore, we show that sAPPα signaling is associated with ERK/MAP kinase signaling. Increased phosphorylation of ERK is indicative of signaling through the MAP kinase pathway. In NPCs, ERK signaling is crucial for carbachol-induced increases in DNA synthesis [41], inhibiting MAP kinase signaling decreases BrdU incorporation after heparin-binding EGF activation [42], and MEK 1 and 2 inhibitor, U0126, inhibits proliferation after hypoxia/reoxygenation [43]. All of these factors point to ERK/MAP kinase as a critical pathway in the proliferation of NPCs. Another critical pathway involved in proliferation of NPCs, Akt [41-44], was not activated by sAPPα *in vitro*. Previous investigations using pharmaceutical inhibitors of either the ERK/MAP kinase pathway or the Akt/PI3-K pathway suggest that PI3-K inhibition results in both self-renewal deficits and proliferation decline whereas MAP-kinase inhibition affected proliferation only [45].

## Conclusions

In summary, we provide evidence that, by signaling through MAP kinase pathways, the evolutionarily conserved sAPPα regulates the proliferation of adult stem cells originating from different germ layers. Future studies should determine whether a reduction in α-secretase activity or in sAPPα production or in both contributes to the aging of these stem cell populations.

## Abbreviations

ADAM: a disintegrin and metalloproteinase; APP: amyloid precursor protein; BCA: bicinchoninic acid; bFGF: basic fibroblast growth factor; BSA: bovine serum albumin; DMEM/F12: Dulbecco's modified Eagle's medium/F12; EDTA: ethylenediaminetetraacetic acid; EGF: epidermal growth factor; ERK: extracellular signal-regulated kinase; hdpPSC: Human decidua parietalis placenta stem cells; MAP: mitogen-activated protein; MMP: matrix-metalloproteinase; MSC: mesenchymal stem cell; NC: negative control inactive inhibitor; NPC: neural progenitor cell; NSC: neural stem cell; PBS: phosphate-buffered saline; PMSF: phenylmethylsulfonyl fluoride; rpm: revolutions per minute; sAPPα: soluble amyloid precursor protein alpha; SGL: subgranular layer of the dentate gyrus; SORLA: sortilin-related receptor with type-A repeats; SVZ: subventricular zone; TBS: Tris-buffered saline.

## Competing interests

The authors declare that they have no competing interests.

## Authors' contributions

MPD carried out experiments and data analysis and participated in manuscript writing.

AB and ZS each collaborated on experiments and participated in experimental design and manuscript writing. OL oversaw studies and participated in study design, data analysis, and manuscript writing. All authors read and approved the final manuscript.
